# Engaging with Peri-Urban Woodlands in England: The Contribution to People’s Health and Well-Being and Implications for Future Management

**DOI:** 10.3390/ijerph110606171

**Published:** 2014-06-12

**Authors:** Liz O’Brien, Jake Morris, Amy Stewart

**Affiliations:** 1 Forest Research, Centre for Ecosystems, Society and Biosecurity, Alice Holt Lodge, Farnham, Surrey GU10 4LH, UK; E-Mail: ejake.morris@forestry.gsi.gov.uk; 2 Forest Research, Northern Research Station, Roslin, Midlothian EH25 9SY, UK; E-Mail: amy.stewart@forestry.gsi.gov.uk

**Keywords:** peri-urban woodland, trees, led and organized activities, affordances, cultural ecosystem services, restoration, co-construction

## Abstract

In this paper we engage with debates concerning people and their contact with the natural environment as part of everyday life drawing on Irwin’s ideas of co-construction and Gibson’s theory of affordances. We focus on peri-urban woodlands in England as important places where people can interact with nature for health and well-being. Qualitative data were collected *in situ* via walks in the woods, focus group discussions and photo elicitation, with a sample of 49 people. These methods provide rich data on the wide range of meanings associated with woodlands that can have a perceived impact on people’s health and well-being. The findings link to contemporary debates about health, well-being and ecosystem services. We explore the inter-play between attributes of the physical environment and the range of facilities provided to enable access, social interactions and the benefits people attribute to their woodland experiences. We conclude that peri-urban woodlands can clearly contribute to self-reported health and well-being in multiple ways, and that organized activities can be important for those who face barriers to accessing woodlands. A strong message emerging from the research is the opportunity afforded by woodlands for social connections with others, as well as the provision of a range of sensory benefits and opportunities to observe and enjoy seasonal change in woodlands. Mental restoration via connection with nature also emerged as important, confirming previous research.

## 1. Introduction

The Millennium Ecosystem Assessment was a major international project that looked at the consequences of ecosystem change for human well-being. The work stated that ecosystem services (provisioning, regulating, supporting, and cultural) are the benefits people obtain from ecosystems [1]. In this conceptualisation human well-being is the key outcome of the benefits derived from ecosystem services. In the United Kingdom (UK) a National Ecosystem Assessment (NEA) was undertaken following a similar approach to the MEA [2]. Cultural ecosystem services in the MEA were defined as the non material benefits obtained from ecosystems and these include well-being benefits such as: spiritual and religious; aesthetic; education; cultural heritage and health. In the UK NEA, Church *et al*. ([3], p. 634) argue that ecosystem cultural services are the environmental settings that have been *co-produced* by people’s constant interactions with nature *i*.*e*., “They [environmental settings] are inscribed with not only natural features but also the legacies of past and current societies, technologies and cultures. The continual change in these settings involves a range of complex cultural practices, such as the development of institutions, the application of capital and human processes involving memories, emotions, the senses and aesthetic appreciation”. The environmental settings identified by Church *et al*. [3] include gardens, formal and informal green and blue spaces and the countryside. In this paper we will focus on the environmental setting of peri-urban woodlands in England as a cultural ecosystem service that can provide health and well-being benefits that are gained via engagement and interaction with this setting.

In the UK the Office for National Statistics was asked by the Government to develop a suite of indicators that capture human well-being beyond gross domestic product, *i*.*e*., to “measure what matters” [4,5]. These indicators fall within a number of domains such as: the economy; education; employment; health; personal well-being; our relationships; where we live; and the natural environment [6]. The “natural environment” domain includes indicators to do with recycling, energy consumption and greenhouse gas emissions. The “where we live” domain provides the only indicator that begins to capture some of the importance of people having contact and interaction with nature for well-being and quality of life. This indicator is currently the percentage of people who have accessed the natural environment at least once a week in the last 12 months [6]. An increasing body of evidence suggests that contact with nature (both green and blue spaces) can be beneficial to people in a variety of ways. The benefits to physical and mental health of contact with nature have been studied widely through for example literature reviews [7,8], primary research [9,10,11,12,13,14,15,16] and meta analyses [17]. The benefits are now also recognized in government health strategies in the UK [18,19,20]. However, some of the benefits gained through active engagement and interaction with nature are described as intangible; such as memorable experiences, spiritual connection, sense of place, and contribution to identify [3,21]. These benefits are seen as less amenable to quantitative assessment, valuation and mapping and are therefore described as more difficult to take into account in decision-making processes and can be left out of valuation studies [3,22,23]. In this research we investigate the relationship between peri-urban woodlands and people’s health and well-being, drawing on the theories of co-construction [24] and affordances [25,26]. Qualitative and/or mixed method approaches such as those used in this research can be important in identifying people’s emotional and spiritual connections to woodlands, and participating in a visit with research respondents is a useful methodological approach as it can provide detailed insights into the meanings and practices people associate with the wooded environment.

## 2. Theoretical Background

How people in the UK engage, describe and identify with trees and woodlands is based on the socio-cultural meanings of trees and woodlands in UK society, representations of trees and woods in literature and contemporary media and art, policies and strategies that enable and encourage, or not, access to woodlands, people’s childhood experiences that are influenced by their parents and peers, and their adult experiences of visiting woodlands alone and / or with friends and family [22,27,28]. Irwin ([24], p. 173) is concerned with society/nature dualism and is keen to move beyond this to “actively generate co-constructions”. The concept of “co-construction captures the dual process of the social and the natural being varyingly constructed within environmentally related practices and particular contexts”. Irwin [24] argues that the society/nature dualism is a barrier to understanding environmental issues and engagement with the environment. While Irwin [24], Hannigan [29] and Yearley [30] talk mainly about the co-construction of environmental problems, in this paper we take Irwin’s idea of co-construction to focus on the co-construction of the meanings of woodlands as an arena for people’s dynamic engagement with nature as part of everyday life and how this contributes to their health and well-being. This involves examining how people talk together about what trees and woodlands mean to them in relation to their experiences and how they engage with trees and woodlands throughout their life. In this conception engagement with woodlands is intertwined with people’s experiences and practices and this is embedded within wider discourses about trees and woodlands in society. The recent conflict generated in 2011–2012 when the UK coalition government published a consultation which proposed the sale of some of the public forest estate (managed by Forest Enterprise) in England illustrates how a catalyst point, such as this, can bring to the fore the meanings and values people hold for trees and woodlands, not normally articulated as part of everyday discourse, and how it can became a topic of debate and discussion across society [31,32].

We also draw on Gibson’s [25] theory of affordances which are the ‘functional properties of an environmental feature for an individual ([26], p. 20). An example of this is a footpath that affords walking opportunities in woodland or a bench that affords the opportunity of resting, picnicking or enjoying a view. In an evaluation of Active England projects in woodlands Morris and O’Brien [33] found that woodland sites that created new play areas for children, and new cycling and mountain bike trails afforded greater opportunities for visitors and changed the profile of those who visited these woodland sites. The concept of affordance concerns the relationship of the person to the environment; this relationship is dynamic and is based on perception and action. People also attach meanings to the environment but also derive meanings from it because of the dynamic way the environment is experienced, how it is perceived, how it is managed and where it is located. For example, if one of the meanings a person associates with woodland, through previous experience, is as a wildlife haven then woodland may afford that person with opportunities to engage with wildlife through bird watching or getting involved in citizen science and monitoring changes in woodland biodiversity. We are interested in how woodlands are experienced by users in the course of their actions and how this contributes to their health and well-being. Heft [26] distinguishes between potential affordances and the affordances that an individual realises through their practices and actions. In this research we are looking at peri-urban woodlands in England situated next to urban areas between the suburbs and the countryside; these are often near to large centres of population. These woodlands provide opportunities for people to connect with nature on a regular basis. The research questions we addressed were: (1) What are the characteristics and components of peri-urban woodland that people regard as influencing their health and well-being and how are these co-constructed via narratives of managed and natural nature, and the special nature of woodlands? (2) What are the range of health and well-being benefits people believe they gain from the affordances they realise while engaging with woodlands, through the activities and practices they undertake?

## 3. Methodology

This study adopted a qualitative research approach and involved the researchers visiting four woodland sites in England (see Table 1) and undertaking a walk or participating in an activity with six groups of research participants lasting approximately one hour (Table 2). Fieldwork was undertaken in May at two of the sites and in September at the other two. Three of the woodlands were chosen for study as they linked to previous research the authors had undertaken in which surveys of woodland visitors and surveys of people living within 3 km of the woods were undertaken to understand woodland visits and the importance of the woodlands for both those who visited and those who did not [34,35,36]. We wanted to supplement this existing work with qualitative data to understand the meanings people associate with these peri-urban woodlands and how it impacts on their health and well-being.

**Table 1:** Details of the four woodland sites in England.

Four Woodland Sites	Shorne Woods Country Park, Kent	Birches Valley, Staffordshire	Bentley Community Woodland, Doncaster	Brodsworth Community Woodland, Doncaster
**Site ownership**	Kent County Council (KCC)	Forestry Commission England (FCE)	Land Trust	Land Trust
**Management of the site**	KCC	FCE	FCE	FCE
**Size of site**	116 hectares	442 hectares	93 hectares	99 hectares
**Woodland type**	Ancient woodland with some heathland, meadows and wetland	Pine plantation and heathland	Mixed deciduous and coniferous woodland with 12 hectare wetland	Mix of mature broadleaf woodland, newly planted trees, large open meadows, wetland valley
**Site designation**	Site of Special Scientific Interest	Part of Cannock Chase Area of Outstanding Natural Beauty	Community Woodland	Community Woodland
**Infrastructure, facilities, opportunities**	Play area, cycling, horse riding, parking, toilets, disabled toilets, fishing, refreshment (café), eco friendly visitor centre, walking trails, trim trail, easy access features for wheelchairs, pushchairs and electric scooters, electro scooter hire, education	Adventure play area, mountain bike trails, horse riding, walking trails, orienteering, Route to health sculpture trail, Go ape, fishing pools, heritage trail, education, visitor centre, refreshments, easy access trail, toilets, disabled toilets, caravan/camp site	Walking, cycling, horse riding, education, picnic tables, miners memorial sculpture, parking, no toilets	Walking, cycling, horse riding, education, orienteering, picnic tables, parking, sculptures, no toilets
**Organised activities, programmes**	Hosts educational programmes and activitiesProvides Forest School	Volunteers, school visits, family events, team building and inset days	Conservation management, health walks and activities	Nordic walking, Green gym, summer activities for children
**Other information**	Green Flag award (the benchmark standard for parks and green spaces in the UK)	Has held pop concerts on site	Former Bentley colliery (coal mine) site	Former Brodsworth colliery site closed in 1990

**Table 2:** Participants involved in the research.

Site	Group Type	Number in Group
Bentley Community woodland	Nordic walkers group	7
	Deaf group	3 deaf people 3 support staff (Sue Ryder staff ^1^ and volunteer FC ranger)
Brodsworth Community woodland	Green gym group (environmental volunteering)	4
Birches Valley Forest Centre	Morning mixed age and gender group	11
	Afternoon mixed age and gender group	8
Shorne Woods Country Park	Jeskyns Wood Volunteer group. The visit took place at Shorne Woods not Jeskyns Wood.	13
**Total participants: 49**		

Note: ^1^ Sue Ryder is a charity organisation providing compassionate care and support to people in need.

The study sites were chosen to provide a distinction between two main groupings:

1 *Local Community Woodlands* (e.g., Bentley and Brodsworth Community Woodlands)—these are sites used by local people who travel from a short distance, who visit reasonably frequently (often every day or every week) and do not stay long at the sites which have no toilets or café. Events and activities may be organised on these sites, however they will be fewer in number than those organised at Destination Woodlands.
2 *Destination Woodlands* (e.g., Birches Valley and Shorne Woods)—these are usually larger in size, used by a mix of local residents and visitors who travel from further afield. People, particularly visitors from further afield, often visit less frequently (a few times a month or a few times a year) but stay longer at the sites which have facilities, such as cafes and toilets. Events and activities will be organised at these sites and they will often get high numbers of visitors, particularly during school and public holidays [36,37].

We specifically wanted to go into and be in nature with people as part of our data collection process. The walk/activity was followed by a focus group discussion, lasting approximately one hour. Photo elicitation was employed with four of the six groups. Two groups did not take photographs as they were carrying out Green gym and Nordic walking activities. The Green Gym is a scheme that involves people in environmental volunteering for health and well-being and Nordic walking is physically active walking with Nordic walking poles. Other participants were asked to take photographs of anything in the woodland that they felt had an impact on their health and well-being. They were asked to write short comments for each photograph, covering: (a) what the photograph was, and (b) what the impact was on their health and well-being. Taking photographs was intended to enable participants to think broadly about the characteristics of the woodland they were in as well as what these meant to them. The focus groups took place after the walks and were used to elicit discussions about the photographs people had taken, and to explore links with health and well-being and current and previous experiences in woodlands. In the focus groups participants were asked to think about the following questions: (1) What are the physical aspects of this site/other woodlands that you think impact on your health and well-being? (2) What are the (social and personal) factors that enable you to access and use this site/other such woodlands for health and well-being benefits? (3) What if anything is the specific contribution of trees and woodlands to health and well-being?

Forestry Commission staff played a vital role in helping the researchers to gain access to existing groups of woodland visitors and in leading the walks. We wanted to include people from a variety of ages and backgrounds who had previously visited the four woodland sites at least once. We had the opportunity of working with a deaf group at Bentley Community Woodland and engaged someone who had expertise in sign language to help us. Participant recruitment was made difficult by the fact that we were asking people to commit approximately 3 hours of their time, a one hour walk, one hour focus group and travel to and from the site. Because of this, some groups were offered the chance to take part in a free organised activity, as well as the research. For example, the two groups at Birches Valley Woodland responded to an advertisement to get involved in the construction of a willow fence for half a day and to take part in our research during the other half of the day. The Green gym (Brodsworth Community Woodland) and Nordic walkers (Bentley Community Woodland) were carrying out their normal activities which the researchers participated in with them. The researchers followed an ethical statement on social research used by social researchers within Forest Research [38] (Social and Economic Research Group, Farnham, UK, 2010). All participants completed consent forms agreeing to be involved in the research, for recording of focus group discussions and for the use of any photographs taken for research purposes and publications.

Demographic data from a short questionnaire showed that out of a total of forty nine people involved in the study, 35% (*n =* 17) were men and 63% (*n =* 31) were women. Not all participants completed all the demographic questions. Just over half (54%, *n =* 25) were in the 45–64 age range. Most were working or retired. All classed their ethnicity as ‘White’. Eight (16%) people stated they were registered as disabled, and two of these were from the deaf group. Seven people (14%) stated that their daily activities were limited significantly by a health problem or disability. Thirteen (26%) said their activities were limited a little by a health problem or disability. Respondents from the four groups that took photographs took a total of 397 pictures. There was a wide variety of photographs and comments illustrating how different people focus on a wide range of factors at each of the sites.

### 3.1. Data Analysis

All of the focus group discussions were recorded. The recordings were fully transcribed and the transcripts and the photographs taken by participants (with the associated notes) were imported and coded using NVivo (a qualitative data analysis software package). The coding was an interpretive process used to organise the qualitative data, it required the researchers to carefully read the data and demarcate segments within it [39]. These segments were then labeled with a code that provided an indication of what was included within that segment. Therefore, the coding process involved the careful reading by the three researchers of each focus group transcript and attributing individual sentences, phrases or words to single or multiple codes and related themes. For example, when exploring participants’ perceptions of the benefits gained from woodland visits a theme emerged that included terms such as calmness, peacefulness, restful and we coded this as “restoration”. We adopted an inductive approach, with codes and themes emerging from careful reading of the transcripts rather than starting deductively with a pre-structured coding schema. Codes were grouped after discussion amongst the research team under four high level themes relating to (1) physical characteristics of the forest; (2) individual health and well-being benefits; (3) social experiences; and (4) the special nature of trees and woods. We have selected quotes and photographs that illustrate the key themes, the name of the group is given after each quote.

## 4. Results and Discussion

We draw on the narratives of respondents to explore the co-constructed meanings of woodlands and the affordances identified and experienced in the four woodlands in our study.

### 4.1. Experiences and Perceptions of the Physical Characteristics of the Woodland Environment

**Figure 1:**
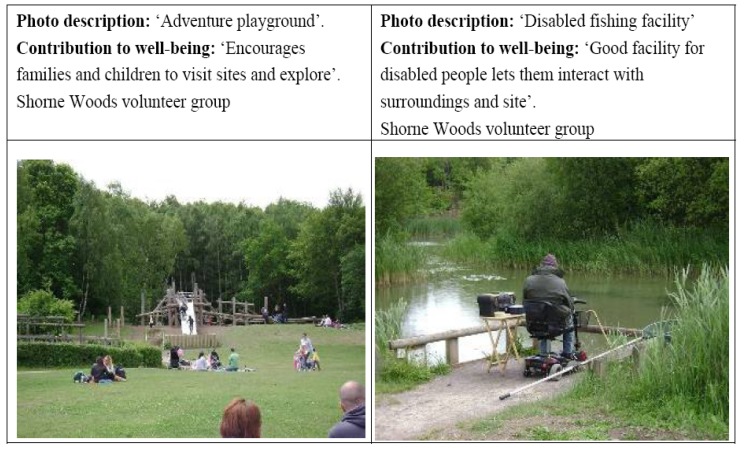
Infrastructure and facilities can attract and assist visitors.

In this theme we focus on the meanings of managed woodland and natural “wild” woodland and how this is shaped by childhood engagement and changed through adult engagement with woodlands and its relation to feelings of health and well-being. In the focus groups participants’ differing co-constructions of wild and natural woodlands were debated, along with the affordances they provide. For those less familiar with visiting and accessing woodlands from an early age, those who were older, and people with mobility problems, woodlands that were managed and had some facilities (e.g., such as good footpaths, toilets, information and directions, benches, car park) were seen as critical in affording practical use and engagement with woodlands. The following quotes and Figure 1 illustrate how particular facilities can afford access and give people the confidence to visit a site. People who were concerned about safety and did not want to visit alone, or were concerned about getting lost also identified the need for higher levels of site management e.g., orientation information, ranger/staff presence or the presence of other users:

*I took some photographs of the logistics*, *if you’ve been ill for a long period of time*, *it’s all very well having this on the doorstep*, *if you can’t access it*, *so I’ve taken photographs of car parks and seats*

(Birches Valley AM group)

But the thing that held me back from visiting was concern that there wouldn't be facilities that I would need such as toilets and the terrain being too difficult for me to walk with confidence

(Birches Valley AM group)

*I took a picture of the bench*, *I’ve sat there with my husband and just sat there peaceful*

(Birches Valley PM group)

The destination woodland sites such as Birches Valley and Shorne Woods were described as places that “*bridge the gap between accessibility and challenge*” (Birches Valley AM group. AM = morning group, PM = afternoon group). These are large enough sites to have facilities (see Table 1) however, there are also areas away from the main car park that only have footpaths rather than wider facilities, and these are quieter with less people accessing them. Parents talked about how they benefited from the infrastructure and equipment that afforded opportunities for their children to play and be physically active, and encouraged and stimulated the children into playing various imaginative games. However, even these groups talked about the need for some areas to have no or little infrastructure and there were interesting discussions about the need to strike a balance between a level of management that afforded various opportunities, and for access that felt and was perceived as wilder and less managed.

Participants used the term unmanaged not to mean neglect but to mean a wood which was constructed as more natural *i*.*e*., it was not obvious that much management was taking place. For some participants, the four woodland sites could act as starting points and after gaining confidence some felt they could branch out further to less managed spaces.

*My dad would prefer to be out in the wild walking on a path that hasn’t been walked in two years or whatever*, *you know? I would be quite able to walk that sort of terrain*, *I would be with him on that*, *you know? Okay*, *I would come to here as a starting point and go off away from all the managed stuff and everything because then you would get into the wildlife*

(Birches Valley AM group)

Quieter places afforded opportunities for improving mental well-being through experiencing peace, reflecting on life and being away from others:

*I’m looking for places where I can go and hide away*, *the old isolation*

(Shorne volunteer group)

Others felt that with careful planning sites that were popular and heavily used such as Shorne Woods and Birches Valley could still offer solitude and escape. Life stage also appears to be an important factor that affects how some people’s preferences, for managed *vs*. natural woodlands, change.

*this is not the kind of place I would choose to come now*, *at the time of my life where I am because I’m young and I don’t have a family and I don’t have children*. *None of the things here interest me but I could see a point in the future where*, *when I do have a family*, *that this would be a lovely place to bring children and it might be something I would use at that stage of my life*, *but where I am now I do want to be away from children essentially*, *I want the quiet and all that*…*I would choose somewhere that was a little bit wilder*

(Birches Valley AM group)

Some participants argued that improvements to health and well-being sometimes required an environment that gave a perceived degree of physical and mental challenge, and that the design of sites and the provision of infrastructure should be informed by all, not just those with specific access requirements.

*You can get someone such as myself in a wheelchair coming [to the site] and going*, *“I can’t do that slope” but if they take the slope out*, *then you're just going through woods and it wouldn't be countryside*, *you can’t have and make everywhere accessible*, *there’s got to be some*...*place where you struggle to get to and that’s half the point of it*, *you know*

(Birches Valley AM group)

**Figure 2:**
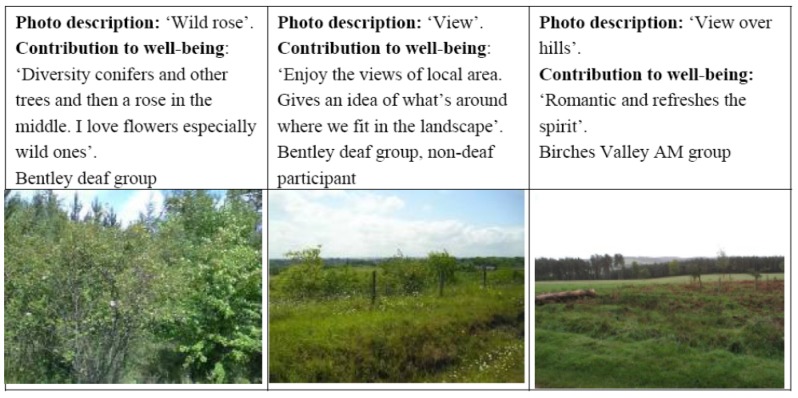
Variety and complexity in the woodland environment.

Our research shows that people display a variety of preferences for trees and woodland, *versus* open green spaces with clear views. Although there is a general sense of appreciation for trees and wooded landscapes (both broadleaved, coniferous and mixed), people also appreciate open spaces with views and clear sightlines. It would therefore appear that many people favour complexity and variety in the landscape, and the sites in this study which were of a reasonable size incorporated a range of habitats such as heathland and wetland, and were able to provide this level of diversity (Figure 2).

### 4.2. Trees and Woods as Special Places?

One of the aims of our research was to address the question of whether trees and woodlands display particular qualities that are significant to people in terms of their perceived health and well-being. Some of the evidence we gathered, associated with sensory experiences, suggests that they do. These qualities, such as sensory experiences and observing changes in the seasons, largely relate to the benefits that individuals perceive they can gain from spending time in woodlands but they are also impacted upon by the physical attributes of the area and how it is managed. For example, aesthetically, participants favoured variety and complexity in the woodland environment including some open spaces that gave them views across the landscape. Our research suggests that the contribution made by trees and woods may, in some respects, be distinct from that made by other flora, habitats, or green space types as trees provide a magnitude of experience due to their scale compared to other vegetation types.

Our research shows that woodlands are richly symbolic environments and that large trees in particular are perceived as the physical expression of a range of meanings and values, many of which were identified by participants as being significant in relation to their percieved health well-being. For example, in terms of the benefits of relaxation, trees helped people to reflect on their problems and life in general as the following quotes suggest.

*For me I think it’s when you look up at a tall tree*, *it makes you feel insignificant somehow*. *But not insignificant bad*, *but insignificant that’s just part of everything*

(Birches Valley PM Group)

*It makes me feel peaceful–insignificant not important—small*, *but makes your earthly worries less fierce*

(Birches Valley AM Group)

When you’re out here you’re thinking of problems on the outside not your own problems

(Bentley Green Gym Group)

*You forget about your worries when you’re out*, *especially with a lung full of fresh air*

(Shorne Woods volunteer group)

The detail of the symbolism attached to trees and woodlands (*i*.*e*., as cathedral like—see below) is likely to be unique in many respects as compared with other components of green space. Some participants referred to the particular qualities of the woodland canopy and how it can engender a sense of safety, security and protection.

You can shelter under them when it rains

(Shorne Woods volunteer group)

*I think there’s strong comparisons with the idea of a tall canopy being majestic*, *similar to a cathedral in its*, *in its proportions and the sense of light playing through the leaves and the branches is similar to that of a stained glass window* ... *And it’s that sense of security that that roof gives you when you’re walking through it*

(Shorne volunteer group)

**Figure 3:**
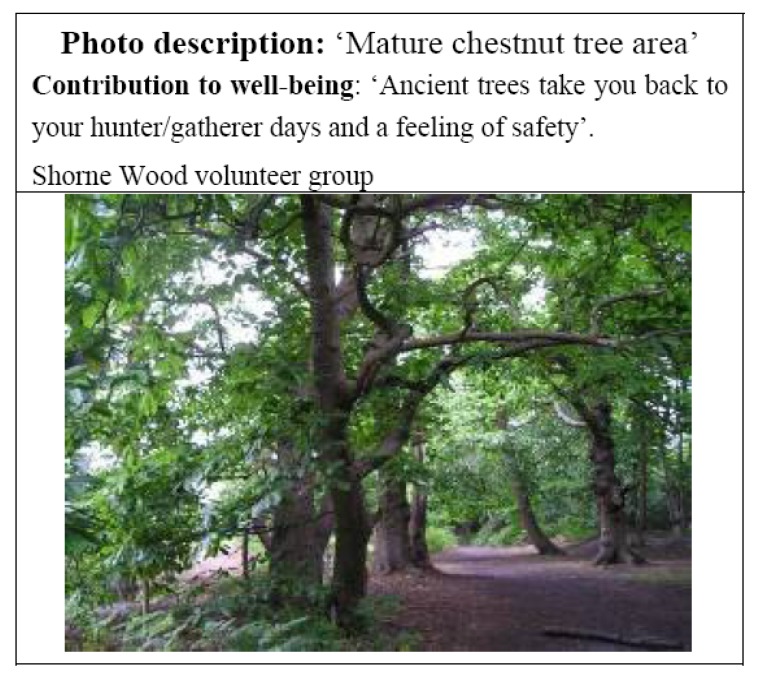
Feelings of security.

Part of the sense of security that trees offer may also be linked to their screening qualities, which some participants also feel helps to create a sense of wilderness and freedom (Figure 3).

*It’s almost that inbuilt childhood thing*. *I don’t know about anyone else but when I was a kid with all my friends we’d go into the woods and the first thing that come to mind was hide and seek and you can just get lost in the woods for hours*. *It’s that ability to just wander off*

(Shorne volunteer group)

Trees also often act as surrogates for wider environmental issues and concerns about nature, particularly the loss or damage to nature in urban environments. Due to their longevity trees give people a symbolic sense of history and continuity with their ability to adapt, regenerate and survive.

*I think it’s that sense of adaptability they have*. *I took a picture of a yew tree and it’s an immeasurably brilliant tree*, *it virtually can’t die*. *They live for ridiculous amounts of years*, *there’s something so noble and charismatic about a big tree*

(Shorne Woods volunteer group)

Broadleaved trees also act as markers of time through the changes they exhibit in response to seasonal variation. For example, changes in leaf colour in the autumn, the loss of leaves in the winter and the new growth, buds and blossom in the spring, with full leaf coverage in summer. Many participants derived perceived well-being benefits from trees’ visual representations of the changing seasons giving a sense of connection to nature cycles and the passing of time. We asked all participants to write down a word or two about their sensory experiences, which can also change through the seasons (Table 3).

**Table 3:** Participants’ examples of sensory aspects of woodlands they enjoyed.

Sensory Experiences	Responses from Participants of Sensory Benefits
Views/aesthetics	*Seeing changes in the seasons*, *the contrast of the tightly compacted trees*, *looking*, *scenery*, *children playing*, *views through the area of woodland*, *view of natural woodland with no man made items in sight*.
Sounds	*Birdsong*, *wind in the trees*, *rustling trees*, *quiet*, *crunchy leaves*, *treading on gravel*, *drowning of traffic noise*, *peaceful*.
Smells	*Smell of damp woods in the autumn*, *smell of pines*, *smell of rotting leaf litter*, *decaying bracken*, *smell of trees and grass*.
Texture	*Bark of trees*, *touching sculptures*, *diversity of textures*, *crunchy stones on path*, *soft grass*.

### 4.3. Individual Health and Well-Being Gained from Engaging with Peri-Urban Woodlands

**Figure 4:**
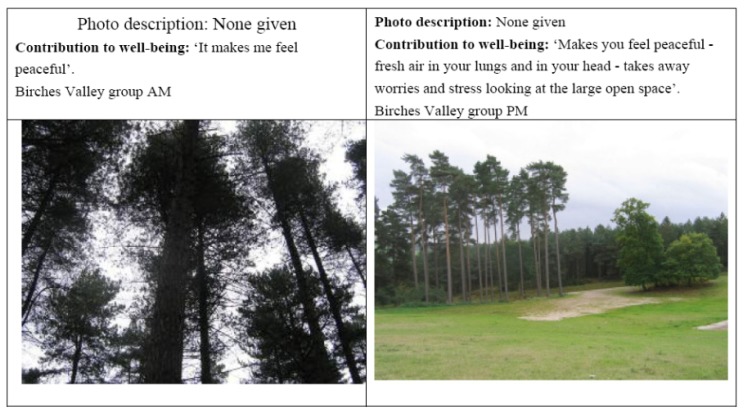
Peacefulness and stress reduction.

Amongst the most common responses from participants concerning benefits of woodland experiences were those alluding to their restorative qualities and their linkages with improved mental and psychological well-being. Participants talked about visits to woodlands as a way of reducing stress, and achieving a state of peacefulness, calm, and restfulness. All the groups involved in the research mentioned some of these aspects (see Figure 4). Some contrasted their feelings of calmness and relaxation in woodlands with being in a town or other kinds of built environment. The concept of restoration could also include stimulation at the same time as relaxation and it could help people who were feeling down or having a bad day. For example, one Birches Valley participant took a picture of the view from a bench saying, “*in all weathers it’s a beautiful view*, *relaxing yet stimulating*”.

*My husband and I will come up here on our own*, *lovely and peaceful*, *not being stressed about anything*, *it’s peaceful and quiet*, *even if there’s a lot of people here it still seems quiet and peaceful*

(Birches Valley AM group)

*When I’ve had a really bad day*, *“I need to go for a walk*, *don't talk to me*, *don’t look at me*, *I’m just not in the mood*, *I’m going to go sit on one of them benches*

(Birches Valley PM group)

Many participants made an association between taking part in an activity in woodlands and feeling healthier both physically and mentally. In particular, they talked about the range of opportunities to be active and exercise as a way of maintaining or improving fitness and/or losing or maintaining weight.

The woodland and tracks make me think I’d like to run round the park for my fitness

(Shorne volunteer group)

It encourages me to come for a walk

(Birches Valley AM)

For one wheelchair user, who was able to use a tricycle at Birches Valley, the all ability trail was particularly important because it provided an even and flat surface, minimising the risk of falling.

*I still use the ‘Route to Health’ [all ability trail] track*, *but I can gauge my fitness on how long it takes me to get round it and then I add another lap or whatever to increase my fitness*

(Birches Valley AM group)

Play spaces and interactive installations could also help to encourage activity in children. One parent noticed that locating the play areas away from the car park could:

Encourage kids to walk to the drums

(Birches Valley AM)

For some participants, getting involved in vigorous activity, such as the Nordic walking or Green gym conservation activities gives them a sense of achievement from undertaking physical exercise. As one participant stated, this kind of physical activity was what the body was designed to do:

*Your body was made to work and it’s like a car*. *If you buy a new car and stick it in the garage it will eventually seize up; this keeps you going*. *I mean I should be in a rocking chair knitting at my age*

(Bentley Nordic walkers group, 75+ age category)

Physical activity was also described as rhythmic by the Nordic walkers, suggesting meditative type benefits:

Participant1: *You get more philosophical*, *getting into a rhythm*
Participant2: Once you get into it you go into almost like a trance
Participant3: *It’s metronomic* (Bentley Nordic walkers group).

Many participants talked about how woodlands signify a connection between themselves and nature. It appears that visits to woodlands have the effect of reinforcing an awareness of this connection by bringing people into direct experiential contact with, and actually being part of, the natural environment and its processes. In turn, this awareness and experience is beneficial in terms of perceived health and well-being generally, and particularly strong in relation to mental health. The following quote from a non-deaf participant in the Bentley deaf group discussions illustrates this:

*It’s sort of a connectedness really when you come out of your home environment outside and particularly wide open spaces or woodlands because you feel yourself moving through that environment and you feel connected to other things around you really*... *I also like foraging in the woods as well so I like the idea that it’s abundant and you can live off it and there are lots of animals and creatures living here*, *this is their world and it’s nice to be a part of that*…*It’s good as well that you appreciate that everything is a living thing and it’s giving out positive energy*... *It makes you feel good because they are giving you energy*. *You just absorb that energy*, *freedom and spirit*.

One person in the Birches Valley afternoon group summed it up by saying: *‘nature is naturally inspiring’*. Some participants talked about acts of reinforcing this sense of connectedness through direct physical contact with trees. These physical acts are overtly communicative and sensory, whereby there is a direct exchange between person and tree.

*I don’t know why*, *I just do*. *I’ve heard of tree huggers but I could*, *I do actually touch the trees in my garden*

(Shorne volunteer group)

For one participant, contact with trees was a cultural practice, opening up a link with previous generations and a time when human beings’ connections with the natural environment were more routinely expressed in everyday practices.

*But trees for humans have always been good because we evolved from monkeys*, *that’s how we first found safety*, *in the trees*. *So there’s got to be some kind of primeval instinct in us*....

(Shorne volunteer group)

During the discussions and in the comments accompanying photographs, participants would often reflect on childhood experiences of visiting woodlands. The evocation of memory and connections between present and past experiences emerged as a strong theme of the research.

*It evokes childhood memories and if you’ve got children and a family of your own*, *you can have a second delight if you like in knowing that whatever you felt or saw yourself you’re sort of having it vicariously again and it’s an absolute pleasure to see that and it makes you feel good*

(Birches Valley AM group)

*It’s like your past perception is how you perceive bliss and happiness is something when you were a child you were free*. *Then you’ve got all the constraints of an adult but you can go back and still get that feeling from looking at a wood or watching other children*

(Shorne volunteer group)

Getting out into the woodland environment gave participants a contrast to their everyday lives and, for some, provided feelings of freedom and escape. This could be escaping from something, such as worries or frustrations, or escaping into a sensory experience. Places with views and vistas were particularly significant in this regard.

*It makes you feel free*, *the wind and the view*

(Bentley deaf group, paraphrased by a non deaf participant)

If you want to think you can and also escape in the view

(Bentley deaf group, non-deaf participant)

This sense of freedom and escape also gave some participants the chance to be themselves, or to just wander through the wood with no specific purpose in mind.

Although the majority of people’s experiences of the behaviour of others in woodlands are positive, our research also highlights some negative dimensions that can act as well-being “detractors”. For example, at Birches Valley parts of a sculpture had been vandalised and one participant took a photograph of this saying it made her unhappy and that she did not understand why it had been done. Another participant from the Shorne volunteer group said that seeing litter made them angry and argued that people should take their rubbish home with them. Indeed litter was highlighted by many participants as representing negative behaviour that they did not want to see and which detracted from their enjoyment of the visit.

### 4.4. Social Experiences in Peri-Urban Woodlands

We were also interested in exploring in more detail people’s social experiences in woodlands, their perceptions of engagement with others in a woodland setting, and how this contributed to their perceived health and well-being. We encouraged discussion within the focus groups around the issues of socialising and visiting with people (e.g., with family, friends or others), visiting alone and getting away from others, and participating in shared activities such as volunteering or group walking. It is possible that social experiences with others were particularly important for many of the participants in this study as some were joining a regular organised activity (e.g., volunteering, Green gym, Nordic walking) or had responded to an advertisement/flyer to get involved in creating a willow fence (Birches Valley participants only). The deaf group in particular stated they were only likely to visit Bentley Community Woodland in a group when they were supported and with someone who knew the site.

There were discussions in the focus groups of the benefits and enjoyment people gained from seeing other people during a woodland visit. This gave many people pleasure, and sometimes enhanced their own experience, making them feel more connected to the woodland, nature and to other people. The sounds others make, such as laughter or children playing can also improve mood.

It makes me smile to see the kids and hear them

(Shorne Woods volunteer group)

I like to see the children’s area because I like seeing the children enjoy their environment

(Shorne Woods volunteer group)

*We saw a man and a horse cantering by*. *It was jolly and makes one feel connected to the community and woodland paths*

(Bentley deaf group)

Seeing others also inspired some participants. For example, one participant was disabled and mainly used a wheelchair but was able to use a tricycle (available for hire at Birches Valley) to ride around the all ability trail. Other participants drew inspiration from this. Sharing a woodland visit with family and friends was important for many participants, as well as having the opportunity to meet new people or to stop and chat to other visitors. For example, a couple stated that visiting with their dogs provided opportunities for socialising:

*We’ve got our dogs and we meet a lot of walking dogs and this last 12 months we’ve made quite a few*, *I won’t say friends but acquaintances*, *other people walking their dogs and you get to chat to them and we look forward to seeing them*…

(Birches Valley AM group)

For some participants, visiting woodlands enabled them to be more sociable and to feel more a part of wider society or their local community thus reducing feelings of social isolation. Taking part in similar activities to other visitors also engenders a sense of affinity with them, for example walking, Go Ape (this is a high wire tree top adventure course), volunteering, or being with children or having a dog. The woodland environment also seemed to act as a space where people felt freer to say hello and speak to strangers than they might otherwise do in other contexts or environments.

*I took a picture of my friend and the people she was talking to because I felt that in terms of your health it’s nice to have gone out somewhere for the day with a friend which makes you feel good*. *But also the fact that you can meet people you don’t know and just chat to them*, *it makes you feel part of society*, *part of the world*. *It’s a better feeling than just staying indoors on your own*

(Birches Valley AM group)

*When we were on that Go Ape thing you talked to them and you didn’t know them but because you’d come to that thing and they’ve come to it as well so you immediately talked to them*. *I’m sure we would talk to people if we were just walking around and they were walking around*

(Birches Valley PM group)

All of the study sites have a programme of organised activities, such as volunteering, nature walks and Nordic walking. The Destination Woodlands (Birches Valley and Shorne Woods) had more regular organised activities than the Local Community Woodland sites (Bentley and Brodsworth). The Birches Valley participants responded to flyers/advertisements to get involved in making a willow fence for half a day, the other half of the day they participated in our research. These types of taster sessions provide people with the opportunity to try something new and to learn a new skill. They also enable people to try something they had previously thought they could not do:

*I think for me it is about when you’ve got a formal day it is about trying new things and then I can take those away with me and then I might re-visit either on my own*, *or I might bring other people and introduce people to the same things*. *It’s about me seeing something new*, *having different ideas*, *being a bit creative*, *just trying to think differently to my normal everyday life*

(Birches Valley AM group)

*Always fancied coming along and it was an opportunity to do that [Nordic walking] without spending on poles and finding out you don’t like it before buying the poles*, *thought I should try it out*

(Bentley Nordic walking group)

For others, participating in led activities provides them with necessary support and encouragement. Some were concerned about getting lost or not knowing where to go, whilst others lacked the confidence to visit alone. This was particularly the case for the deaf participants, who stated that they would prefer to visit the site with support:

*If I come*, *I have a support worker now 3 times a week*, *I would come with her or probably with people but I wouldn’t come on my own I fear*…

(Bentley deaf group)

*I wouldn’t know my way around so*, *no*, *I wouldn’t [visit alone]*. *It’s better in a group because you get guided around the woodland*

(Bentley deaf group)

*I think it’s better with other people*. *J says it’s much more enjoyable if you’re in a group and also there is individual help available*

(Bentley deaf group)

Participant’s also derived benefits from seeing others engaging with and behaving in woodlands in a positive way. For example, one participant talked about seeing families together enjoying themselves in the woods and described this as an important social expectation *i*.*e*., that the parents were introducing their children to nature.

*It’s nice to see parents with children*, *walking their children around because they’re obviously bringing them up in the right way*

(Birches Valley AM group)

## 5. Discussion

We have found from this and previous research that people attach a wide range of meanings to trees and woodlands and value them for many reasons [37,40,41,42]. The experiences people have in woodlands provide them with a variety of perceived health and well-being benefits that can vary with the different woods they visit, the types of activities they undertake as well as the social aspects of their visits. The links between the various personal, social and physical characteristics we have identified in this research do not have distinct boundaries or fall into neat categories as the context of a specific visit to a particular woodland is important in terms of people’s motivations and expectations of health and well-being benefits. This research identifies a range of cultural well-being benefits, which are a key concept of the ecosystems identified in the UK NEA [3]. Our approach of being *in situ* in woodlands with participants provided an important methodological tool to explore people’s relationships with woodlands in depth. We could observe how the participants were engaging with the woods through the walk and as they took their photographs. For example, some participants stopped to chat to other people in the woods such as dog walkers, they talked to each other and watched others in the woods as well. The photo elicitation provided people with a novel opportunity to consider how the pictures they took represent anything about the woodlands that influenced their health and well-being. Woodlands are multi-functional and synergistic satisfiers *i*.*e*., they can satisfy a number of different needs at the same time [3]. The concepts of co-construction and affordance are useful in studying the dynamic relationships between people and woodlands. We have outlined the various meanings people co-construct in their on-going engagement with trees and woodlands, we also show how particular environmental features can afford opportunities for different groups that can contribute to their health and well-being. A strong message emerging from the research, providing new insights, is the importance of the social connections with others that many people experience in woodlands. This occurs through people not only socializing with friends or family or saying hello to strangers in the woods, but also through enjoying and benefiting from watching others, such as families. Seeing people behave in positive ways in woodlands was perceived as beneficial, however seeing evidence of negative behaviours such as discarded litter and vandalism reduce people’s enjoyment. The special nature of trees and woodlands, such as shelter, natural grandeur and providing a visual representation of seasonal change and natural cycles, gives people an opportunity for reflection and to connect with what matters to themselves. These findings resonate with other research where people talk about a simpler life in conjunction with connection to nature [43]. This research also provides a confirmation of the opportunity afforded by woodlands for mental restoration, which has been found in much previous research [7,8].

In terms of woodland management the research highlights the need to understand the access requirements of the current and potential users of a particular site. Those with health and mobility problems and the elderly have specific needs in terms of access infrastructure and facilities on site in order to make a visit feasible. Gathering such information is important for woodland managers to inform site design. All of the sites in the study run organised events and activities. Some are led by Forestry Commission England staff, and some by staff from other organizations that are using these public sites to work with a range of groups, or by volunteer groups. What the research highlights is the importance of these led and organised activities and events, particularly for those who are less familiar with visiting woodlands, those who do not want to visit alone, those keen to meet other people or try out a different activity, and those who have concerns about getting lost. Organised activities can also be important for reaching people at different life stages, such as those who are retired, children with their parents, and the unemployed. Events can attract new woodland users and the size of the sites, such as those studied in this paper, means they can absorb a number of visitors and activities without seeming overcrowded. Careful targeting of activities may be needed to reach and attract particular groups that face barriers to accessing woodlands [44]. Recurring activities such as health walks or volunteering can attract those who want to visit on a regular basis adding structure to their lives and maybe important for those who may want to become more familiar with accessing woodland.

It was clear from our research that participants undertook a range of behavioural strategies in order to be able to access the woodlands and gain perceived health and well-being benefits. For example, those who were concerned about getting lost or needing support would only visit with others, women who were concerned about being alone only visited with friends and family. Those who wanted to relax focused on visiting the quieter areas of the woodlands, while those who wanted to socialize came with family, friends or joined an activity or event. These varying strategies can potentially affect how often people will visit woodlands *i*.*e*., you may have to wait for others to be free so that you do not visit alone. Understanding the current users and potential users of the woodlands is important and requires understanding of the catchment population that lives within a few kilometers of a woodland site, as well as visitors from further afield.

We are also aware of some of the limitations of our study for example all four of the woodland sites are of a reasonable size *i*.*e*., over 93 hectares which meant that some facilities were available at each site (Table 1), the sites are all open for public access and access is actively encouraged. It is less likely that the wide range of benefits gained by participants would be available on sites with limited or restricted access, or on very small woodland sites. The sample was not ethnically diverse, we originally aimed to include those who rarely visited woodlands (which might include ethnic minorities), however this proved difficult because of the ‘*in situ*’ method we used. Future research with those who never or rarely visits woodlands might be more successful if it was conducted in a local community rather than in a woodland setting.

## 6. Conclusions

There is an existing body of evidence that outlines the range of health and well-being benefits people can gain from accessing woodlands and greenspace [7,8,9,10,11,12,13,14,15,16,17]. Our research adds to this body of evidence by focusing specifically on peri-urban woodlands near to large centres of population. Some previous research highlights that social experiences in woodlands can be important and our research adds further insights into this. However, further research is needed to understand this social dimension of woodland visits for different age groups and across the life course. Further work is needed to understand the most effective forms of intervention to promote the health and well-being benefits of urban and peri-urban forests amongst different social groups. A current government focus is how to influence and potentially change people’s behaviours so that they can adopt more sustainable and healthy lifestyles. There are a range of factors that enable, mediate, or restrict the realisation of well-being benefits from trees, woods and forests e.g., well-being is the outcome of different configurations of and interactions between the physical woodland or tree resource, governance structures and processes, the characteristics of individuals or groups of beneficiaries and the different activities they participate in. Woodlands that are designed for and promote healthy living provide important opportunities to a wide range of people for improving health and well-being and connecting people to nature.

## References

[1] Millennium Ecosystem Assessment (2005). Ecosystems and Human Well-Being: Synthesis.

[2] UK National Ecosystem Assessment (2011). The UK NEA: Synthesis of the Key Findings.

[3] Church A., Burgess J., Ravenscroft N. (2011). Cultural Services: Chapter 16. UK NEA.

[4] Department for Environment, Food and Rural Affairs (2007). Sustainable Indicators in Your Pocket 2007. An Update of the UK Government Strategy Indicators.

[5] Office for National Statistics Measuring What Matters. http://www.ons.gov.uk/ons/guide-method/user-guidance/well-being/index.html.

[6] Office for National Statistics Measuring National Well-Being: Life in the UK. http://www.ons.gov.uk/ons/rel/wellbeing/measuring-national-well-being/first-annual-report-onmeasuring-national-well-being/art-measuring-national-well-being-annual-report.html.

[7] Croucher K., Myers L., Bretherton J. (2007). The Links between Green Space and Health: A Critical Literature Review.

[8] Hartig T., Mitchell R., de Vries S., Frumkin H. (2014). Nature and health. Annu. Rev. Public Health.

[9] Kaplan S., Relf D. (1992). The Restorative Environment: Nature and Human Experience. The Role of Horticulture in Human Well Being and Social Development.

[10] Berman M.G., Jonides J., Kaplan S. (2008). The cognitive benefits of interacting with nature. Psychol. Sci..

[11] Hartig T. (2008). Green space, psychological restoration, and health inequality. Lancet.

[12] Mitchell R., Popham F. (2008). Effect of exposure to natural environment on health inequalities: An observational population study. Lancet.

[13] Hug S.-M., Hartig T., Hansmann R., Seeland K., Hornung R. (2009). Restorative qualities of indoor and outdoor exercise settings as predictors of exercise frequency. Health Place.

[14] Kessel A., Green J., Pinder R., Wilkinson P., Grundy C., Lachowycz K. (2009). Multidisciplinary research in public health: A case study of research on access to green space. Public Health.

[15] Maas J., Verheij R.A., de Vries S., Spreeuwenberg P., Schellevis G., Groenewegen P.P. (2009). Morbidity is related to a green living environment. J. Epidemiol. Commun. Health.

[16] Nordh H., Grahn P., Wahrborg P. (2009). Meaningful activities in the forest, a way back from exhaustion and long term sick leave. Urban For. Urban Green..

[17] Bowler D., Buying-Ali L., Knight T., Pullin A. The Importance of Nature for Health: Is There a Specific Benefit of Contact with Green Space?. http://www.environmentalevidence.org/SR40.html.

[18] Department of Health (2011). Start Active, Stay Active: A Report on Physical Activity for Health from the Four Home Countries’ Chief Medical Officers.

[19] Department of Health (2011). No Health Without Mental Health: A Cross Government Mental Health Outcomes Strategy for People of All Ages.

[20] Department of Health Change for Life–Eat Well, Move More, Live Longer. http://www.nhs.uk/Change4Life/Pages/change-for-life.aspx.

[21] Natural England (2009). Experiencing Landscapes: Capturing the Cultural Services and Experiential Qualities of Landscape.

[22] O’Brien E. (2005). Publics and woodlands: Well-being, local identity, social learning, conflict and management. Forestry.

[23] Fish R., Burgess J., Church A., Turner K. (2011). Shared Values for the Contributions Ecosystem Services Make to Human Well-Being.

[24] Irwin A. (2001). Sociology and the Environment.

[25] Gibson J. (1979). The Ecological Approach to Visual Perception.

[26] Heft H., Ward Thompson C., Aspinall P., Bell S. (2010). Affordance and the Perception of Landscape: An Inquiry into Environmental Perception and Aesthetics. Innovative Approaches to Researching Landscape and Health.

[27] Ward Thompson C., Aspinall P., Montarzino A. (2008). The childhood factor: Adults visits to green places and the significance of childhood experience. Environ. Behav..

[28] Tabbush P. Cultural Values of Trees, Woods and Forests. http://www.forestry.gov.uk/pdf/SERG_Cultural_values_of_trees_research_summary.pdf/$FILE/SERG_Cultural_values_of_trees_research_summary.pdf.

[29] Hannigan J. (1995). Environmental Sociology: A Social Constructionist Perspective.

[30] Yearley S. (1992). The Green Case: Sociology of Environmental Issues, Arguments and Politics.

[31] (2012). Independent Panel on Forestry. Final Report.

[32] BBC Forest Sale Axed: Caroline Spelman Says “I’m Sorry”. http://www.bbc.co.uk/news/uk-politics-12488847.

[33] Morris J., O’Brien E. (2011). Encouraging healthy activity amongst under-represented groups: An evaluation of the Active England woodland projects. Urban For. Urban Green..

[34] Morris J., Doick K. Annex 1: Flagship Case Study Report: Bentley Community Woodland. Monitoring and Evaluating Quality of Life for CRS07. http://www.forestry.gov.uk/pdf/CSR07_Bentley_Community_Woodland_2009-10.pdf/$FILE/CSR07_Bentley_Community_Woodland_2009-10.pdf.

[35] Morris J., Doick K., Cross D. (2011). The Contribution of Trees, Woods and Forests to Quality of Life: An Evaluation of Quality of Life at Three Case Study Sites.

[36] Morris J., Doick K. Annex 2: Flagship Case Study Report: Birches Valley Forest Centre. Monitoring and Evaluating Quality of Life for CRS07. http://www.forestry.gov.uk/pdf/CSR07_Birches_Valley_final_0809.pdf/$FILE/CSR07_Birches_Valley_final_0809.pdf.

[37] Molteno S., Morris J., O’Brien L. (2012). Public Access to Woodlands and Forests: A Rapid Evidence Review.

[38] SERG SERG: Research Ethics. Forest Research, Farnham. http://www.forestry.gov.uk/pdf/SERG_Statement_of_Research_Ethics_SEPT_2010.pdf/$FILE/SERG_Statement_of_Research_Ethics_SEPT_2010.pdf.

[39] Braun V., Clarke V. (2006). Using thematic analysis in psychology. Qual. Res. Psychol..

[40] Bell S., Hamilton V., Montarzino A., Rothnie H., Travlou P., Alves S. (2008). Green Space and Quality of Life: A Critical Literature Review.

[41] O’Brien L., Williams K., Stewart A. Urban Health and Health Inequalities and the Role of Trees, Woods and Forests in Britain: A Review. http://www.forestry.gov.uk/pdf/SERG_Urban_health_and_forestry.pdf/$FILE/SERG_Urban_health_and_forestry.pdf.

[42] O’Brien L., Morris J. (2013). Well-being for all? The social distribution of benefits gained from woodlands and forests in Britain. Local Environ..

[43] Christmas S., Wright L., Morris L., Watson A., Miskelly C. Engaging People in Biodiversity Issues. http://randd.defra.gov.uk/Default.aspx?Module=More&Location=None&ProjectID=18411&FromSearch=Y&Publisher=1&SearchText=WC1056&SortString=ProjectCode&SortOrder=Asc&Paging=10.

[44] Morris J., O’Brien L., Ambrose-Oji B., Lawrence A., Carter C. (2011). Access for all? Barriers to accessing woodlands and forests in Britain. Local Environ..

